# A randomized pilot and feasibility trial of live and recorded music interventions for management of delirium symptoms in acute geriatric patients

**DOI:** 10.1186/s12877-025-05954-1

**Published:** 2025-05-02

**Authors:** Jelena Golubovic, Bjørn Erik Neerland, Melanie R. Simpson, Kjersti Johansson, Felicity A. Baker

**Affiliations:** 1 Centre for Research in Music and Health (CREMAH), Norwegian Academy of Music, Oslo, Norway; 2 Oslo Delirium Research Group, Department of Geriatric Medicine, Oslo University Hospital, Oslo, Norway; 3 Department of Public Health and Nursing, Norwegian University of Science and Technology, Trondheim, Norway; 4 Faculty of Fine Arts and Music, The University of Melbourne, Melbourne, Australia

**Keywords:** Feasibility, Delirium, Music, Music therapy, Severity, Covid-1

## Abstract

**Background:**

Delirium is an acute shift in attention and arousal, usually triggered by acute illness or surgery in older dementia patients. Prognosis is poor, and pharmacological options are limited; non-pharmacological interventions and music show promise.

**Methods:**

This randomised pilot and feasibility trial tested feasibility, acceptability, fidelity, and safety of music interventions (MIs) for delirium patients and assessed preliminary effectiveness and suitability of the selected effect outcomes. Participants from an acute geriatric ward were randomised to Preferred Recorded Music (PRM) and Preferred Live Music (PLM), delivered for 30 min over three consecutive days. Feasibility outcomes included recruitment rate, retention, adherence, deviations, and treatment fidelity. Clinical outcomes were trajectory of delirium symptoms (arousal, attention, cognition), delirium duration, hospital stay length, and medication intake. Post-intervention and between groups changes in delirium symptoms were compared using mixed linear regression models for the repeated measurements. Mann-Whitney test and Fishers exact test were used for length of stay and medication use, respectively.

**Results:**

26 participants (PLM = 14; PRM = 12), median age 87, most with hypoactive delirium were recruited at a rate of three participants per month. Retention rates for PLM and PRM were 64% and 33% respectively and adherence to PLM and PRM intervention protocols were 83% and 58%, respectively. Total adherence to the assessment protocols was 44%. PLM was delivered as intended, (treatment fidelity 93%), and PRM did not satisfy treatment fidelity (83%). All delirium symptoms except arousal improved on day 3 compared to baseline, with statistically significant improvement in attention. No conclusive pre-post or between-group differences were detected for any outcomes; confidence intervals were wide.

**Conclusions:**

Feasibility of recruitment, interventions and assessments was indicated, and greater acceptability, safety and fidelity of the PLM intervention compared with the PRM. Adoption of external assessors is warranted in future trials, to mitigate slow recruitment and low adherence. Wide confidence intervals for most measures and comparisons indicate that the possible effect of the MIs on delirium cannot be excluded. The trial was registered at Clinical Trials, ID: NCT05398211, on 31/05/2022.

**Supplementary Information:**

The online version contains supplementary material available at 10.1186/s12877-025-05954-1.

## Background and objectives

Delirium is a clinical syndrome represented by an acute change in mental status [1] and inattention; cognitive dysfunction, disturbed level of arousal, psychotic episodes, emotional and behavioural changes may also be present [1, 2]. Delirium is common in hospital settings, occurring in > 50% of patients [3], with the highest prevalence in older, frail [4–6], acutely hospitalized, and mechanically ventilated patients in postsurgical intensive care units (ICUs) [7–9]. Delirium develops suddenly, is transient with fluctuating symptoms [7]. Pathophysiology is complex and not completely understood, involving an interplay between the predisposing factors such as advanced age, underlying illness like dementia, and precipitating factors such as acute illness, drugs, or surgery [7].

Prognoses is poor, including prolonged hospitalization [10], need for long term care [11, 12], onset or worsening of cognitive impairment [7, 10], and increased mortality risk [7, 13]. Non-pharmacological, multicomponent approaches might address multifactorial delirium etiology, and are showing promising effects in preventing delirium [3, 13], decreasing agitation [8, 14], and deriving interest, pleasure and general well-being [15]. The evidence of their effectiveness in clinical management of delirium symptoms is still scarce [14, 16].

Music interventions (MIs) have positive effects on behavioural and psychological symptoms of conditions similar to delirium, such as dementia [17–24] and disorders of consciousness [25, 26], and may also be effective in prevention and treatment of delirium in older individuals. Our systematic review [27] showed that research on MIs for delirium is scarce; existing trials show promising results, particularly for delirium prevention. High patient acceptability and enjoyment of MIs, cost-effectiveness, and absence of adverse effects were also commonly reported [27]. Despite the moderate-high risk of bias among the included studies, our meta-analysis indicated that postsurgical, critically ill, and mechanically ventilated patients were 50% less likely to develop delirium after being exposed to music postoperatively [27]. Significant improvements in engagement, mood, and delirium severity were also found in acute geriatric and long-term care patients post-intervention [28–30]. However, better designed trials, with standardized interventions, in specific clinical settings and utilizing outcome measures able to capture clinically meaningful changes, are still needed to substantiate existing effectiveness claims. The aim of the current trial was to pilot test and establish feasibility to determine the need for investing in a future randomised controlled trial (RCT) design. The objectives of this study were to establish: (1) the feasibility of recruitment procedures and establish the likely recruitment rate; (2) the feasibility of assessments and follow-up procedures; (3) the success of and fidelity adherence of interventions implemented by the therapist; (4) the interventions’ acceptability; (5) the safety of the interventions; and (6) the sensitivity and suitability of the selected effect-outcomes to assess the efficacy of the music interventions. Clinical objectives were: (1) to estimate preliminary efficacy of live and recorded MIs on severity of delirium symptoms, and (2) to establish preliminary evidence of the specific delirium symptom domains most responsive to the MIs.

## Methods

### Trial design

We adopted a two-arm randomized repeated measures design to evaluate the feasibility and preliminary effectiveness of Preferred Recorded Music (PRM), and Preferred Live Music (PLM) interventions. Our study protocol detailing the intervention description, theoretical rationale, and statistical analysis plan, was published prior to the completion of data-collection [31]. This trial is reported in accordance with the Consolidated Standards of Reporting Trials (CONSORT) statement’s extended checklist for pilot and feasibility trials (Additional file 8) [32]. The trial was registered at Clinical Trials (NCT05398211) on 31/05/2022.

### Participants

Participants were recruited from an acute geriatric ward within the Division of Geriatric Medicine at Oslo University Hospital (OUH), where the prevalence of dementia and delirium is high. The 20 bed ward admits 75-80 older patients per month (≥65 years) for acute medical care [33]. After delirium screening at admission, using 4 AT [34]—a rapid validated tool for delirium detection—patients with the score ≥ 4 were assessed by geriatricians for eligibility. Delirium was diagnosed according to the Diagnostic and Statistical Manual of Mental Disorders, 5 th Edition (DSM-5) criteria [1] applying a recommended diagnostic test battery, previously described in our protocol [2, 31, 35–38] (Additional file 2). Subtypes were determined using the well validated Delirium Motor Subtyping Scale (DMSS-4) [39].

Patients were eligible if:

1. aged ≥ 65 years
2. diagnosed with delirium or subsyndromal delirium within the last 72 hours and still present
3. informed consent was obtained.

Patients with comorbidities such as dementia, mild cognitive impairment, or those under long-term care were also included, and we did not exclude patients with COVID-19. Patients were excluded if:

1. Previously enrolled in the study and were readmitted to the ward during the study period
2. Presenting with severe hearing impairments
3. Presenting with severe psychiatric conditions other than delirium
4. Admitted due to severe alcohol or substance addiction
5. Their assessed musical preferences included orchestral or other kinds of music impossible to perform live by voice and accompaniment

The 5th criteria was included after the trial commenced, registered at Clinical Trials on the 5th of December, 2022, and included in the published protocol [31].

### Interventions

Two interventions tested in this study were 1) Preferred Recorded Music (PRM) and 2) Preferred Live Music (PLM). PLM included live music delivered by voice and a guitar with improvisation elements; the PRM involved pre-recorded music delivered via a loudspeaker.

The potential therapeutic efficacy of preferred music, underlying both interventions, stems from its personal, social and cultural attributes, allowing it to alter emotional responses [40], boost self-awareness [41], and transiently enhance certain cognitive functions like autobiographical memory [42]. The live music component and responsive musical and non-musical interactions between the patient and the intervention facilitator in the PLM intervention were expected to regulate arousal levels and attention. Synthetic, loudspeaker sound and original versions of the preferred music in PRM intervention were expected to stimulate autobiographic memory and moderate attention and arousal. A more detailed description of the interventions and the theoretical rationale is provided in our published protocol [31].

Both interventions were delivered by a certified music therapist (MT) who also designed the interventions.

After the baseline assessments, the MT assessed participants’ music preferences from legal guardians, using an adapted Norwegian version of the Assessment of Personal Music Preference tool (APMP) [43], and after that, directly from the participants in an interactive, 30 min’ session, using a Music Assessment Tool (MAT) [44]. Music for the interventions was selected by the MT using the acquired information. The participants received their allocated interventions once a day, for 30 min (between 8 AM and 5 PM) over three consecutive days, in addition to usual care. Participants were considered to have adhered to the intervention protocol if they completed at least 10 min of the sessions.

MIs were primarily delivered in the participants’ private rooms, except when they shared a room with another patient.

### Outcomes

Our published protocol previously described outcomes relevant to our feasibility and clinical objectives and the detailed assessment schedule [31]. The main properties of the assessment tools are provided in Additional files 1–3.

Feasibility outcomes were assessed during and upon the completion of the intervention period and comprised:

1. Recruitment rate: an average number of patients recruited per month.
2. Retention rate: the proportion of participants completing the study as described in the protocol. *Withdrawals* were defined as withdrawing consent to participate in the study. *Dropouts* were defined as any discontinuations of the interventions and assessments due to the participants’ health condition, discharge, or an unavailable assessor. *Refusals* were defined as the patient declining invitations to be involved in assessment or treatment.
3. The proportion of sessions where the MIs and pre-post assessments were completed as planned (adherence to study protocol) and the proportion of sessions with protocol deviations. Deviations were categorized as patient or interventionist-related and further classified as minor, major, or fatal based on their impact on data quality and patient safety, with the fatal category indicating patients’ death [45].
4. The success of treatment fidelity (TF) was determined by observing the video recordings of 20% of randomly selected participants from both intervention groups who had completed the interventions as per protocol. Video recordings were evaluated by an independent rater using a bespoke checklist for each intervention. The six checklist items were scored and calculated (no = 0, yes = 1 point), and the threshold for satisfied treatment fidelity for each participant was ≥ 80% averaged across the three intervention days, including satisfied compulsory items 4-6 for each session. The intervention was considered not to have met fidelity if the compulsory items were not satisfied even if the total score was ≥ 80%.

Secondary clinical outcomes were assessed at baseline, within one hour pre- and post-session, and at discharge by the specially trained geriatricians at the ward and included:

1. Trajectories of delirium symptoms, assessed using DSM-5 diagnostic test-battery [31] comprising: Observational Scale of Level of Arousal (OSLA) [36] and modified Richmond Agitation Sedation Scale (mRASS) [46–48] for level of arousal; backwards tests and digit span tests for attention [35, 38].; orientation and short-term memory, using delayed recall tasks and orientation questions from Memorial Delirium Assessment Scale (MDAS) [49] for orientation and short term memory.
2. Duration of delirium: determined by an experienced delirium researcher (BEN) after the discharge from the ward, based on all the previously assessed data.
3. Length of hospital stay (LOS)
4. Use of Pro Re Nata (PRN), non-prescribed psychopharmacological medication (benzodiazepines and antipsychotics).

LOS and use of psychopharmacological medications during the hospitalization were retrieved from the electronic medical journals at discharge (Additional file 1).

Adverse events were recorded after music and assessment sessions and from daily reports by the medical staff at the ward. The events were categorized as critical and non-critical for the patients’ health and well-being and related or unrelated to the interventions.

### Sample size

This feasibility trial did not intend to draw conclusive findings on the effects of MIs on delirium symptoms, and did not need to be adequately powered. Initially aimed to recruit 60 participants, 30 in each arm, to obtain sufficient data to examine the main objectives of the study, while allowing for potential dropouts.

### Randomization

Eligible, consenting patients were randomized to study arms using permuted block randomization 1:1, and the online randomization software, True Random Numbers (https://www.random.org/). An independent researcher generated randomization blocks of 10 participants, to maintain even number in the two study-arms. The participants were assigned to their respective interventions by the music therapist, after baseline delirium assessments and before assessments of music preferences.

### Masking

The therapist and the participants could not be masked to group allocation due to the nature of the interventions; assessors were masked. To increase the success of masking of assessors, staff at the ward were instructed not to reveal what treatment arm participants were assigned to, and the music therapist walked into the participants’ rooms with her guitar and the loud-speaker, regardless of assigned intervention.

### Statistical methods

The statistical analysis plan is described in our previously published protocol [31].

Linear mixed models were used to estimate the change in OSLA, mRASS, attention and cognitive status from pre- to post-intervention and from baseline for each assessment day, and for the comparison between the intervention groups. The marginal effects were calculated for each of these comparisons, with adjustments for the participants'baseline scores, and using participants’ ID as a random effect, to incorporate the individual differences into the analysis. The Mann-Whitney test for skewed data was used to calculate the difference between the groups in length of hospital stay. We applied Fisher’s Exact test for small samples to determine group differences in received PRN medication.

## Results

### Participants flow and recruitment

**Fig. 1:**
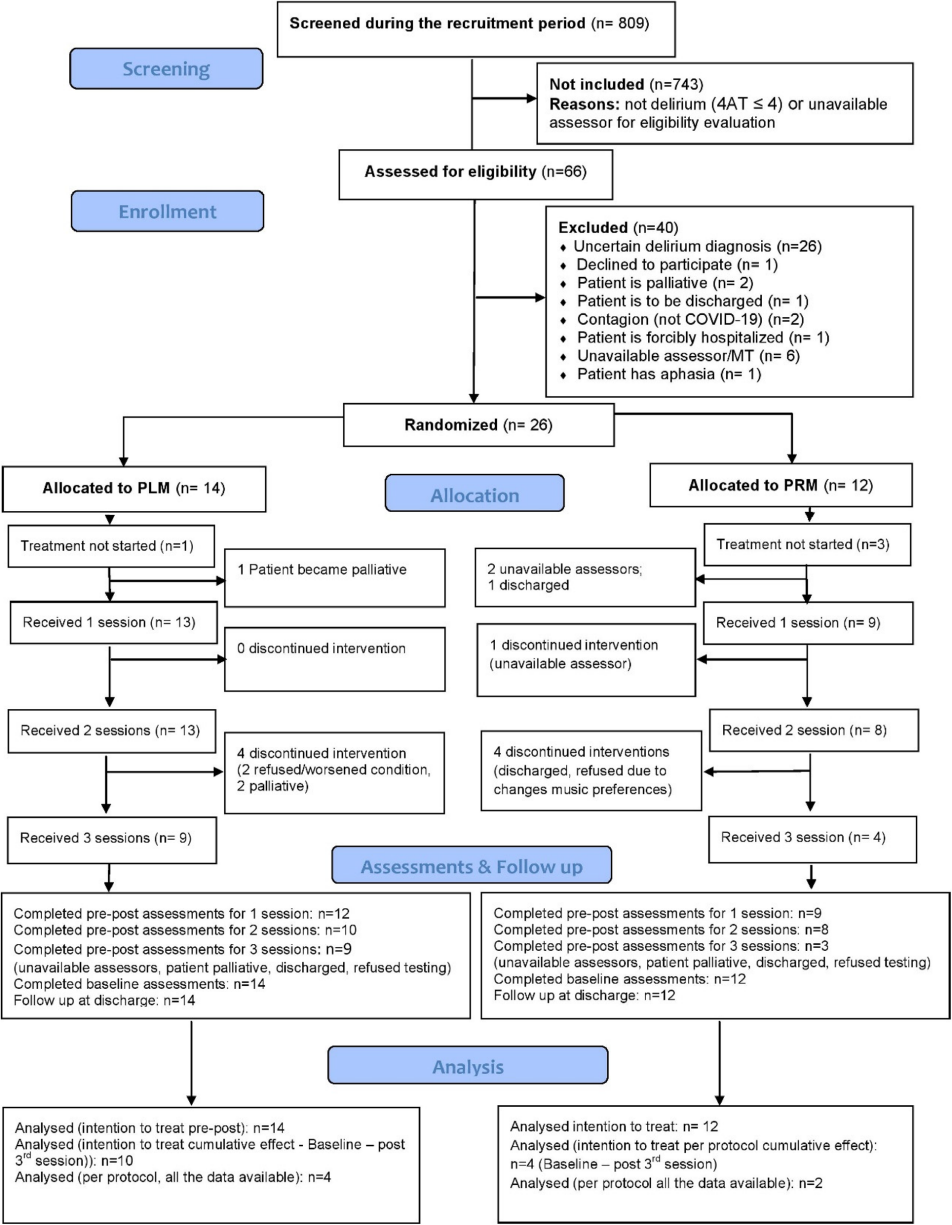
CONSORT flow chart

Potential participants were screened for eligibility between 15th June 2022 and 21st April 2023 (approximately 39 weeks). Of the 809 patients admitted to the acute geriatric ward during the recruitment period, 66 patients were assessed for eligibility (Fig. 1). Of these, 40 were excluded due to uncertain delirium diagnosis, patient receiving end of life care, or being discharged before the sessions could begin, contagion (other than COVID-19), aphasia preventing completion of assessments of delirium, unavailable assessor for the rest of the evaluations, or music therapist unavailability. In total *n* = 26 patients met all the inclusion criteria and were randomized (PLM, *n* = 14; PRM, *n* = 12) (Fig. 1).

Baseline demographic and clinical characteristic were similar across the intervention groups (Table 1). Participants’ median age was 87, half were female (*n* = 13), and the majority lived alone (*n* = 19, 73%,), or with others (*n* = 4, 15%,) in the community. All had DSM-5 delirium at baseline, 68% hypoactive and 20% had no recognizable subtype. In 73% of cases (*n* = 19), infection, fracture or a cardiovascular event precipitated delirium. For some participants the trigger was unknown, or hard to ascertain due to underlying dementia or depression. Clinical frailty scale scores were obtained for *n* = 15 participants (58%), showing scores of ≥ 5 in 14 out of 15 tested patients, and a median score of 7. Along with the median frailty index of 0.40 in these participants, this score indicated severe frailty. The median NEWS2 score of 3 at admission to the AG ward indicated low to moderate acute illness severity (Table 1).

**Table 1:** Demographic and clinical characteristics at baseline

Characteristics	All participants *n* = 26	PLM *n* = 14	PRM *n* = 12
Demographics			
Age, median (IQR)	87.0 (80.8–92.3)	87.0 (79.3–92.3)	89.5 (83.3–92.8)
Women, n (%)	13 (50)	7 (50)	6 (50)
Men, n (%)	13 (50)	7 (50)	6 (50)
Place of residence before hospitalization			
Private home, with others, n (%)	4 (15.4)	4 (28.6)	0
Private home, alone n (%)	19 (73.1)	10 (71.4)	9 (75.0)
Assisted Living facility, n (%)	2 (7.7)	0	2 (16.7)
Residential care home, n (%)	1 (3.8)	0	1 (8.3)
Level of care			
No public services, n (%)	5 (19.2)	2 (14.3)	3 (25.0)
House help/practical assistance, n (%)	1 (3.8)	1 (7.1)	0
Home nursing care, n (%)	16 (61.5)	8 (57.1)	8 (66.7)
Long term residential care, n (%)	2 (7.7)	1 (7.1)	1 (8.3)
Other, n (%)	2 (7.7)	2 (14.3)	0
Medical, at admission			
Reasons for hospitalization, n (%)			
Fall, fracture, injury, n (%)	12 (46)	3 (21)	9 (75)
Chest pain, shortness of breath, n (%)	4 (15)	1 (7)	3 (25)
Delirium, confusion, somnolence, n (%)	8 (30)	3 (25)	5 (42)
Other, n (%)	9 (35)	9 (64)	0
Clinical Frailty Scale, median (IQR)^a^	7.0 (6.0—7.0)	7.0 (5.0—7.0)	6.0 (6.0—7.0)
Frailty index (FI), median (IQR) ^e^	0.4 (0.4—0.5)	0.4 (0.3—0.4)	0.4 (0.4—0.5)
Number of prescribed medications, median (IQR)	5.5 (3.0—7.0)	6.0 (3.5—8.3)	5.0 (3.0—6.0)
IQCODE ^b^	3.6 (3.3—4.5)	3.4 (3.0—3.4)	3.7 (3.5—3.7)
NEWS II at admission to hospital, median (IQR)^c^	3.0 (1.0—6.0)	3.0 (1.0—5.8)	3.5 (0.3—6.8)
NEWS II at admission to the acute geriatric ward	3.0 (1.0—5.0)	2.0 (0.8—5.3)	3.0 (2.3—5.0)
Delirium status at baseline			
DSM-5 delirium, n (%)	26 (100)	12 (100)	14 (100)
Digit Span forward, digits correct, median (IQR)	3.0 (0.0—4.3)	3.00 (0.8—4.3)	3.5 (0.0—4.8)
SAVEAHAART/KATAMARAAN, number of mistakes, median (IQR)	2.0 (0.0—10.0)	3.5 (0.8—10.0)	1.0 (0.0—8.0)
Days of the week backwards, numbers correct, median (IQR)	3.0 (0.0—7.0)	1.0 (0.0—6.3)	5.5 (0.0—7.0)
Months of the year backwards, numbers correct, median (IQR)	3.0 (0.0—5.5)	1.5 (0.0—4.3)	4.5 (0.0—7.0)
Count backwards from 20 to 1, numbers correct, median (IQR)	7.0 (0.0—20.0)	2.5 (0.0—15.5)	14.5 (3.5—20.0)
Orientation, number of correct items, median (IQR)	3.0 (0.8—7.0)	2.5 (0.0—4.8)	5.5 (2.3—7.0)
Delayed recall, numbers correct, median (IQR)	0.0 (0.0—0.0)	0.0 (0.0—0.0)	0.0 (0.0—0.8)
OSLA score, median (IQR)	2.5 (1.3—6.8)	2.0 (1.5—8.0)	3.0 (1.0—6.0)
mRASS score, median (IQR)	0.0 (−1.0—0.0)	0.0 (−1.0—0.5)	−0.5 (−1.7—0.0)
Delirium motor subtype, n (%) according to DMSS4 ^d^			
Hyperactive, n (%)	2 (8)	1 (8)	1 (8)
Hypoactive, n (%)	17 (68)	10 (77)	7 (58)
Mixed, n (%)	1 (4)	1 (8)	0
No subtype, n (%)	5 (20)	1 (8)	4 (33)

*PLM* Preferred Live Music, *PRM* Preferred Recorded Music, *IQR* Inter quartile range, *IQCODE* Informant Questionnaire on Cognitive Decline in the Elderly, *CFS* Clinical Frailty Scale, *NEWS2* National Early Warning Score II, *DSM-5* Diagnostic and Statistical Manual of Mental Disorders, Fifth Edition, *SAVEAHAART/KATAMARAN* Vigilance test, *OSLA* Observational Scale of Level of Arousal, m*RASS* Modified Richmond Agitation Sedation Scale, *DMSS4* Delirium Motor Subtype Scale 4

^a^Clinical frailty scale missing in 11 patients; 4 in PLM group and 7 in PRM group

^b^IQCODE missing in 20 patients; 11 in PLM group and 9 in PRM group

^c^NEWS2 missing in 2 patients in PLM group

^d^DMSS4 missing in 1 patient in PLM group

^e^ Frailty Index missing in 3 patients

### Feasibility outcomes

The average recruitment rate was 3 participants per month (Table 2). The retention rates for the participants in PLM and PRM groups were 64% and 33% respectively. Most withdrawals were due to worsening of the participants’ health condition, but none withdrew their consent to use the data collected prior to withdrawal.

**Table 2:** Feasibility outcomes

Measure	Total (*n* = 26)	PLM group (*n* = 14)	PRM group (*n* = 12)
Recruitment rate	3/month		
Participants who completed the study (Retention), n (%)	13 (50)	9 (64)	4 (33)
Participants who discontinued the study (Attrition), n (%)	11 (42)	3 (21)	8 (67)
Intervention sessions completed (Adherence to interventions) n (%)	56 (72)	35 (83)	21 (58)
Pre-post assessments completed (Adherence to assessments), n (%)	34 (45)	26 (62)	18 (50)
Participants who completed the study procedures as per protocol ^a^ (Adherence to study protocol), n (%)	6 (23)	4 (29)	2 (17)
TF (%) ^b^	NR	93% ^c^	83% ^d^

*PLM* Preferred Live Music group; *PRM* Preferred Recorded Music group, *TF* Treatment Fidelity

^a^Patients who completed interventions and assessments as per protocol

^b^Treatment Fidelity average success rate per condition

^c^Compulsory items 4–6 in checklists are satisfied

^d^Compulsory item 6 in checklists was not satisfied

The total adherence to the study protocol (both interventions and assessments) was 29% in the PLM (*n* = 4 patients), and 17% in the PRM group (*n* = 2 patients). Five additional participants in the PLM group met the per-protocol criteria but were excluded because their assessments occurred outside of the assessment window.

The total number of conducted music sessions, including those with deviations, was 55 out of the planned 78 (PLM = 42; PRM = 36). Common reasons for missing sessions were: 1) minimum dosage of 10 min was not met, 2) unavailability of assessor/music therapist to assess/treat, 3) patient became palliative, or 4) patient was discharged. The percentage of adherence to the PLM and PRM protocols were 83% and 58% respectively. Of the 21 completed PRM sessions, 17 were delivered with some deviations (81%), of which the most common were the unwanted interaction between the patient and the therapist, and in some cases changing the order of songs from one intervention day to another. No significant protocol deviations were registered during the PLM, and most sessions were delivered as intended.

The success of TF was 93% for the PLM, including satisfied complementary items 4-6 in the checklists, and 83% for the PRM, with one of the complementary items not satisfied. As planned, music preferences were assessed with input from the legal guardians and participants. Only two participants were not able to contribute to music preference assessments due to their worsened health condition at the time of assessment (Table 2).

Of the planned 78 pre-post delirium assessments in both groups, 34 were successfully completed (44%). Adherence to the assessment procedures in PLM was 62%. Ten pre-post assessment were not completed due to unavailable assessor, patient discharged or becoming palliative: six of the pre-post assessments were out of the assessment window. Adherence to the assessment procedures in PRM was 50%, with 15 pre-post assessments missing due to the unavailable assessor, patient discharged or becoming palliative, and three pre-post assessments were conducted outside of the assessment window.

One serious adverse event (death) occurred during the intervention period but was unrelated to the interventions. Minor, potentially intervention-related events such as patients falling asleep while listening to music, as well as showing signs of boredom and restlessness or wanting to switch faster to the next song, were recorded during the PRM sessions.

### Secondary clinical outcomes

In addition to its within-subject focus, this two-arm trial compared the interventions to determine which might be more suitable for a future definitive trial. With no control group, the comparative analysis remained exploratory and complemented the primary feasibility assessments. An intention-to-treat principle was used to analyse efficacy outcomes, such that the analysis included also those participants who were discharged prior to completion of the three music sessions, or were not able to receive the interventions as per protocol for other reasons. We also intended to complete a per-protocol analysis, but with few participants completing the interventions as described we ultimately opted not to undertake this analysis.

Within-subject analyses (pre-post).

Comparing baseline and pre-intervention scores showed that delirium symptoms varied during the first three days of the intervention, with measures of level of arousal, attention, orientation and short-term memory fluctuating for most individuals. On average, the participants’ level of arousal was similar across the three assessment days. However, their performance on attention tests improved on day 3 comparing baseline and pre-intervention scores, and was statistically significant for counting from 20 to 1, weekdays backwards, digit span memory test and SAVEAHAART vigilance tests (Table 3).

**Table 3:** Daily mean score for clinical delirium outcomes and change from baseline (for the entire sample)

Measure ^b^	Day	Mean (95% CI) ^a^	Mean difference (95% CI) ^a^	*p*-value
OSLA	Baseline	4.2 (3.1 to 5.3)	Ref	
	Day 1	4.4 (3.3 to 5.5)	0.1 (−1.3 to 1.4)	0.930
	Day 2	4.9 (3.7 to 6.1)	0.3 (−1.1 to 1.7)	0.659
	Day 3	3.4 (2.0 to 4.8)	−0.6 (−2.3 to 1.1)	0.502
mRASS	Baseline	−0.6 (−0.9 to −0.3)	Ref	0
	Day 1	−0.8 (−1.2 to −0.5)	−0.2 (−0.7 to 0.3)	0.379
	Day 2	−1.0 (−1.4 to −0.6)	−0.3 (−0.8 to 0.2)	0.189
	Day 3	−0.4 (−0.9 to 0.0)	0.2 (−0.4 to 0.7)	0.546
Count 20 to 1	Baseline	8.7 (5.8 to 11.6)	Ref	0
	Day 1	9.5 (6.4 to 12.6)	0.7 (−2.7 to 4.2)	0.687
	Day 2	9.3 (6.1 to 12.6)	0.5 (−3.1 to 4.1)	0.776
	Day 3	13.9 (10.2 to 17.6)	4.8 (0.5 to 9.0)	0.027
Days of the week	Baseline	3.2 (2.2 to 4.3)	Ref	
	Day 1	4.0 (2.9 to 5.1)	0.7 (−0.6 to 1.9)	0.287
	Day 2	3.5 (2.3 to 4.6)	0.2 (−1.1 to 1.5)	0.779
	Day 3	5.4 (4.1 to 6.8)	2.1 (0.6 to 3.6)	0.006
Months of the year	Baseline	3.1 (1.8 to 4.5)	Ref	
	Day 1	2.7 (1.2 to 4.1)	−0.6 (−2.1 to 1.0)	0.493
	Day 2	3.3 (1.8 to 4.9)	0.2 (−1.5 to 1.8)	0.825
	Day 3	3.3 (1.6 to 5.1)	−0.1 (−2.1 to 1.8)	0.882
Digit span	Baseline	2.8 (2.1 to 3.5)	Ref	
	Day 1	4.0 (3.3 to 4.7)	1.2 (0.4 to 2.0)	0.003
	Day 2	3.2 (2.5 to 4.0)	0.5 (−0.3 to 1.4)	0.205
	Day 3	4.4 (3.6 to 5.3)	1.7 (0.8 to 2.7)	0.001
SAVEAHAART	Baseline	4.1 (2.9 to 5.4)	Ref	
	Day 1	3.6 (2.3 to 5.0)	−0.5 (−1.9 to 1.0)	0.523
	Day 2	3.6 (2.2 to 5.0)	−0.7 (−2.2 to 0.9)	0.398
	Day 3	1.8 (0.2 to 3.3)	−2.3 (−4.1 to −0.6)	0.010
Orientation	Baseline	3.4 (2.5 to 4.3)	Ref	
	Day 1	3.8 (2.9 to 4.7)	0.5 (−0.6 to 1.6)	0.355
	Day 2	3.6 (2.6 to 4.6)	0.2 (−0.9 to 1.3)	0.699
	Day 3	4.8 (3.6 to 5.9)	1.2 (−0.1 to 2.5)	0.073

*OSLA* Observational Scale of Level of Arousal, m*RASS* Modified Richmond Agitation Sedation Scale *SAVEAHAART/KATAMARAN* Vigilance test

^a^Marginal means and mean differences estimated using mixed linear model

^b^Recall is not presented in this table because linear regression was not suitable (see Additional file 5)

There was no significant change in delirium symptoms pre to post MIs on each day. Analysing the data without considering the specific intervention day or group but still considering that each person had up to seven measures did not show significant change either (Table 4). The individual trajectories showed that some symptoms for given participants did change from pre to post MIs, but these changes were in the negative direction. The residuals for delayed recall score were so skewed that linear modelling of this outcome was not appropriate and we instead calculated the proportion of the participants who could successfully recall at least one word. The total percentage of the participants who could recall at least one word on the delayed recall test was only 19% (95% CI: 8.2–38.9) (Additional file 5).

**Table 4:** Before and after music intervention each day (for the entire sample)

Measure ^b^	Day	Before	After	Mean difference (95% CI)^ a ^	*p*-value
OSLA	1	4.4 (3.3 to 5.5)	3.7 (2.6 to 4.9)	−0.6 (−2 to 0.8)	0.414
	2	4.9 (3.7 to 6.1)	3.1 (1.9 to 4.4)	−1.4 (−2.9 to 0.2)	0.077
	3	3.4 (2 to 4.8)	3.5 (2.0 to 4.9)	−0.2 (−2.2 to 1.8)	0.837
	Any ^C^	4.2 (3.3 to 5.0)	3.5 (2.6 to 4.4)	−0.6 (−1.6 to 0.3)	0.206
mRASS	1	−0.8 (−1.2 to −0.5)	−0.7 (−1 to −0.3)	0.2 (−0.3 to 0.7)	0.401
	2	−1 (−1.4 to −0.6)	−0.6 (−1 to −0.2)	0.3 (−0.2 to 0.8)	0.238
	3	−0.4 (−0.9 to 0)	−0.6 (−1 to −0.1)	−0.1 (−0.7 to 0.6)	0.805
	Any	−0.8 (−1 to −0.5)	−0.6 (−0.9 to −0.4)	0.1 (−0.2 to 0.5)	0.378
Count 20 to 1	1	9.5 (6.4 to 12.6)	8.7 (5.5 to 11.9)	−0.3 (−3.9 to 3.4)	0.889
	2	9.3 (6.1 to 12.6)	11.7 (8.3 to 15.2)	2 (−2 to 5.9)	0.332
	3	13.9 (10.2 to 17.6)	10.9 (6.9 to 14.9)	−3.2 (−8.3 to 2)	0.228
	Any	10.6 (8.2 to 13.1)	10.4 (7.9 to 12.9)	−0.2 (−2.7 to 2.4)	0.904
Days of the week	1	4 (2.9 to 5.1)	4.1 (2.9 to 5.3)	0.2 (−1.1 to 1.5)	0.744
	2	3.5 (2.3 to 4.6)	4.6 (3.4 to 5.8)	1.1 (−0.4 to 2.5)	0.142
	3	5.4 (4.1 to 6.8)	4.7 (3.4 to 6.1)	−0.8 (−2.6 to 0.9)	0.339
	Any	4.2 (3.3 to 5.1)	4.5 (3.5 to 5.4)	0.3 (−0.6 to 1.1)	0.538
Months of the year	1	2.7 (1.2 to 4.1)	3.0 (1.5 to 4.5)	0.5 (−1.1 to 2.2)	0.526
	2	3.3 (1.8 to 4.9)	4.7 (3.1 to 6.3)	1.5 (−0.3 to 3.3)	0.104
	3	3.3 (1.6 to 5.1)	4.2 (2.3 to 6.0)	0.9 (−1.4 to 3.3)	0.435
	Any	3.1 (2 to 4.3)	4.0 (2.8 to 5.2)	1.0 (−0.1 to 2.1)	0.080
Digit span	1	4.0 (3.3 to 4.7)	3.3 (2.6 to 4.1)	−0.6 (−1.5 to 0.3)	0.171
	2	3.2 (2.5 to 4.0)	3.8 (3.0 to 4.6)	0.5 (−0.4 to 1.4)	0.294
	3	4.4 (3.6 to 5.3)	3.7 (2.8 to 4.5)	−0.9 (−2.0 to 0.3)	0.139
	Any	3.8 (3.3 to 4.4)	3.6 (3.0 to 4.2)	−0.3 (−0.8 to 0.3)	0.357
SAVEAHEART	1	3.6 (2.3 to 5.0)	4.0 (2.6 to 5.4)	0.1 (−1.4 to 1.7)	0.869
	2	3.6 (2.2 to 5.0)	3.5 (2.1 to 5.0)	−0.1 (−1.7 to 1.6)	0.915
	3	1.8 (0.2 to 3.3)	2.1 (0.5 to 3.7)	0.3 (−1.7 to 2.3)	0.797
	Any	3.1 (2.1 to 4.2)	3.3 (2.2 to 4.4)	0.1 (−0.9 to 1.1)	0.874
Orientation	1	3.8 (2.9 to 4.7)	3.5 (2.5 to 4.5)	−0.4 (−1.6 to 0.7)	0.476
	2	3.6 (2.6 to 4.6)	4.5 (3.4 to 5.5)	0.8 (−0.4 to 2.1)	0.190
	3	4.8 (3.6 to 5.9)	4.2 (2.9 to 5.4)	−0.5 (−2.1 to 1.1)	0.559
	Any	4.0 (3.3 to 4.8)	4 (3.2 to 4.8)	−0.1 (−0.8 to 0.7)	0.894

*OSLA* Observational Scale of Level of Arousal, *mRASS* Modified Richmond Agitation Sedation Scale *SAVEAHAART/KATAMARAN* Vigilance test

^a^Marginal means and mean differences estimated using mixed linear model

^b^Recall is not presented in this table because linear regression was not suitable

^c^“Any” lines combine information from all days, across the intervention groups, taking into account that each person has up to 7 measures

Group comparison.

There was no evidence of a difference between the participants’ delirium symptoms in PLM or PRM groups on day 3 of the intervention (Additional file 4). Group differences on days 1 and 2 were not examined on the assumption that the potential difference in the effect of the interventions would be most relevant on day 3. The participants’ average length of hospital stays in PLM was 11 days (SD = 8.95) and in PRM 13 days (SD = 8.94); there was no statistically significant group difference between the PLM and PRM (*U* = 82.500, *p* = 0.94). The results of Fisher’s exact test did not indicate statistically significant difference between the number of patients receiving PRM medication in the two intervention groups (Additional file 6). There was no sufficient data in the medical journals to estimate changes in delirium duration.

## Discussion

### Feasibility outcomes

This feasibility study demonstrated that implementing PLM and PRM with vulnerable delirium patients at the AG ward was feasible and that the MT could successfully conduct music preference assessments. Obtaining music preferences from the legal representatives prior to direct, interactive patient assessments provided a familiar reference point for dialogic method and recognizing musical examples, thereby forstering a personalized setting that activated recognition memory and facilitated further retrieval of musical memories [43]. However, interactive assessments may have influenced delirium outcomes before the music interventions (MIs) began, introducing a potential confound. They may also have generated expectations regarding subsequent interventions and affected test performance.

Consistent with prior findings identifying hypoactive delirium as the most prevalent subtype [44, 50], 68% of our sample exhibited hypoactive delirium. Because distinct delirium subtypes present different symptoms and warrant tailored interventions, previous recommendations have encouraged investigating interventions separately for each subtype [51]. This recommendation aligns with our suggestions for further investigation into MIs.

PLM demonstrated robust adherence, high TF, a relatively high retention rate, consistent dosage delivery, and minimal protocol deviations, indicating strong feasibility and acceptance. No adverse events or unusual treatment effects were recorded, and the lack of refusals further supports its safety. Descriptive data from the music therapist’s session notes and checklists also suggest PLM was engaging, with participants singing, moving, or reminiscing in nearly all sessions. Notably, 77% of participants in the PLM arm had hypoactive delirium, highlighting the potential relevance of PLM for this particular subtype.

PRM showed lower adherence, retention, and inconsistent session durations (10–33 min). Discontinuations were mainly due to declining health, palliative status, or discharge—rather than refusals—suggesting these findings may not reflect low acceptability. However, therapist notes indicated that patients were actively engaged in only half of the sessions, often exhibiting restlessness, a desire to skip songs, or requesting to end sessions early. Such responses imply PRM may be less engaging and potentially monotonous, likely due to its non-interactive delivery. This observation aligns with earlier assumptions that prolonged loudspeaker-based music can lead to habituation and boredom in delirium patients [31, 52].

Although PRM achieved an 83% success rate, it did not meet TF criteria due to repeated breaches of the protocol’s prohibition on patient-therapist interaction. These interactions—always patient-initiated regarding confusion, pain, distress, or a desire to discuss the music—were ethically justifiable but diminished treatment consistency. Excluding the music therapist (MT) from the delivery altogether could address this issue, though it raises patient-safety concerns about unsupervised music exposure. Alternatively, replacing the MT with another health practitioner would render any interaction typical of usual care.

Recruitment and assessment procedures proved feasible and accurately identified eligible delirium patients. However, reliance on internal assessors—available only two weekdays during day shifts—limited recruitment due to high workloads and other responsibilities. For future research, employing external assessors who can be present most shifts, seven days a week, is advisable to enhance recruitment efficiency.

The recommended test battery for the pre-post assessments was efficient, accurate in assessing delirium and its features, and suitable for application at the AG ward. However, the completion time varied among the assessors. As there is currently no definitive diagnostic test for delirium, its detection depends on assessing key features, combining observation, cognitive testing, patients’ medical history and clinical interviews [2]. By integrating continuous (symptom-based) and dichotomous (delirium yes/no) measures, our harmonized test battery offers more nuanced insights into the trajectory of delirium severity and contributes to the development of more robust, reliable, and standardized delirium assessments in the future [2]. This test battery was also well received by delirium patients, as refusals were rare and typically attributable to significant clinical deterioration. Protocol deviations were minimal, primarily involving delayed or omitted assessments. The lack of clear post-intervention effects may suggest that the one-hour assessment window was too lengthy, potentially obscuring more immediate impacts. Future studies could address this by employing more flexible external assessors who can align measurement schedules more closely with the interventions’ end.

Although the test battery showed high accuracy, efficiency, and suitability, overall adherence to the three-day, multi-measure follow-up protocol was low. However, this was largely due to assessor unavailability and patients becoming palliative or being discharged, suggesting that low adherence may not reflect the feasibility of the follow-up protocol itself. Some participants exhibited substantially worse post-intervention performance on certain cognitive or attention tasks. While the music interventions might have contributed, repeated testing could also be to blame. Although the three-day protocol generated extensive data, it may have overburdened this vulnerable patient group, potentially causing exhaustion, fatigue, or boredom and thus affecting test outcomes. Consequently, future research should carefully re-evaluate the repeated-measures design and the length of the follow-up period.

### Clinical outcomes

Our results showed that the participants’ performance on the attention tests improved significantly on day three, when comparing baseline and pre-interventions scores, while most of their other symptoms were similar to baseline. However, without a control group, the observed changes are difficult to attribute to the MIs delivered the previous day, as delirium usually is usually reversed by treatment of underlying causes [7, 53]. Nevertheless, the summary evaluation of individual DSM-5 criteria showed that most participants still had delirium at the end of the intervention period.

No statistically significant pre-post intervention changes or inter-group changes in delirium symptoms were observed for any of the measures. However, the trial was underpowered to detect preliminary effectiveness properly. Accordingly, the CIs for mean differences were wide for most measures, and we cannot exclude the possibility of changes in delirium symptoms associated with the interventions. The percentage of participants who could recall at least one word on the delayed recall tests was very low. With small samples in addition, testing pre-post intervention and between the groups, change in proportion would have provided no conclusive findings and was omitted. No significant differences in LOS and intake of PRN medication between the groups were detected as the study was underpowered to provide conclusive findings in this regard, and changes in these measures could be correlated with many other factors. The follow-up of delirium duration after the intervention period was unsuccessful due to the transient and fluctuating delirium nature, making it difficult to ascertain whether it had been recovered.

Despite not showing sensitivity to the MIs, clinical outcomes tested in this trial are still highly relevant for detecting changes in delirium progression and severity and should be included in the future. However, to capture the potential effects of the MIs, other complementing outcomes, such as biomarkers, patient-centred outcomes (emotional responses and engagement), or environmental outcomes related to the medical ward and staff, should be considered and explored. Data from the MT’s session notes and checklists indicated that relevant intervention-related changes might also have occurred during the MI sessions, and it is recommended that future trials consider assessing these changes more systematically.

Testing clinical outcomes did not provide sufficient information to discern which of the two MIs could more effectively impact delirium symptoms. The wide CIs are mainly associated with small samples but indicate that the potential effect of PLM and PRM interventions cannot be ruled out.

### Strengths

The trial was sufficiently powered to assess feasibility outcomes, addressing a noted gap in music and delirium research, where methodological quality is often low to moderate and feasibility evaluations are frequently overlooked [27]. Key strengths include the use of validated and recommended delirium assessment methods [2, 35, 37, 38], targeted assessor training, and involvement of an experienced delirium researcher to final interpretation of the findings. Subtyping delirium is another strength, given its clinical and prognostic importance [54–56]. Furthermore, employing a detailed, theoretically grounded intervention protocol with treatment fidelity assessments enhances the generalizability of future efficacy findings [27]. Lastly, involving a trained music therapist (MT) and conducting music preference assessments allowed interventions to be personalized and potentially safer, in line with existing recommendations [27].

### Limitations

Despite being adequately powered to examine feasibility, the trial lacked sufficient power to investigate preliminary efficacy or compare between groups, where a minimum sample size of 30 per group is recommended [57]. No control group was included due to the primary feasibility focus and uncertainties related to delirium diagnosis and recruitment. The reliance on on-site staff during regular working hours slowed recruitment and led to missed assessments, as staff managed competing responsibilities. Although delirium subtyping was incorporated, the limited sample size precluded separate analyses for hypoactive and hyperactive subtypes, which likely require different management approaches. Future research should address these constraints by including a control group, considering distinct delirium subtypes, and improving recruitment strategies, for instance, by engaging dedicated external assessors.

## Conclusion

The feasibility of recruitment procedures, music preference assessments, MIs and assessment protocols were indicated and the results showed that PLM intervention is more engaging, better accepted, and potentially more suitable for further testing with acutely ill older patients with delirium. Recommended next steps are to undertake a pilot study with a comparative group, assess preliminary efficacy, estimate the size of the treatment effects, and to explore different intervention dosages and frequency of delivery.

## Supplementary Information

Additional file 1. Assessment tools and their properties.

Additional file 2. Diagnostic algorithm for DSM-5 delirium evaluation.

Additional file 3. Checklists for treatment fidelity evaluation (PLM, PRM).

Additional file 4. Group PLM versus Group PRM difference on Day 3.

Additional file 5. Proportion of participant who could recall any words in delayed recall test.

Additional file 6. Between the groups difference in Length of hospital stay and PRN medication.

## Data Availability

The datasets may be provided by the corresponding author on reasonable request.

## Abbreviations

AG: Acute Geriatrics
APMP: Assessment of Personal Music Preference tool
CAM-ICU: Confusion Assessment Method for the Intensive Care Unit scale
CI: Confidence Interval
CONSORT: Consolidated Standards of Reporting Trials
CSF: Clinical Frailty Scale
DMSS-4: Delirium Motor Subtyping Scale
DOWB: Days of the Week Backwards
DSM-5: Diagnostic and Statistical Manual of Mental Disorders, 5th Edition
FI: Frailty Index
ICU: Intensive Care Unit
IQCODE: Informant Questionnaire on Cognitive Decline in the Elderly
LOS: Length of stay
MAT: Music Assessment Tool
MDAS: Memorial Delirium Assessment Scale
MI: Music Interventions
MOYB: Months of the Year Backwards
mRASS: Modified Richmond Agitation Sedation Scale
MT: Music Therapist
NEWS2: National Early Warning Score 2
OSLA: Observational Scale of Level of Arousal
OUH: Oslo University Hospital
PLM: Preferred Live Music
PRM: Preferred Recorded Music
PRN: Pro Re Nata, non-prescribed psychopharmacological medication
SAVEHAART: Ten-letter vigilance “A” test
TF: Treatment Fidelity
