# Supplementary material for: A randomized pilot and feasibility trial of live and recorded music interventions for management of delirium symptoms in acute geriatric patients
Source: BMC Geriatr. 2025 May 2;25:306. doi: 10.1186/s12877-025-05954-1 (PMC12048927; doi:10.1186/s12877-025-05954-1)
Supplement: Supplementary file 1 — Additional file 1. Assessment tools and their properties. [file 12877_2025_5954_MOESM1_ESM.docx]

**Additional file 1.** Assessment tools and their properties

| Type of data | Assessment Tools | Scale-Properties/Cut-off |
| --- | --- | --- |
| Sociodemographic and clinical background data (assessed at baseline) | | |
| Age  Gender  Place of residence  Level of medical care before hospitalisation  Past and current medical conditions  Prescribed medication | Electronic medical journals | N.R. |
| Pre-admission cognitive status | IQCODE: Informant Questionnaire of Cognitive Decline | The short version of the validated Informant Questionnaire on Cognitive Decline in the Elderly. Consists of 16 questions scored from 1-5, with a score of ≥ 3.3 indicating worsening (49) |
| Frailty status | CFS: Clinical Frailty Scale | A nine-point scale, evaluations based on the descriptions of functional status and activity, and scores ranging from 1 (very fit) to 9 (terminally ill) (50). |
|  | FI: Frailty index | Scores below 0.12 indicate the person is fit; higher scores indicate greater frailty (0.12-0.24, mildly frail, 0.24 to 0.36, moderately frail, 0.36 and above, severely frail) (51). |
| Severity of acute illness | NEWS2: National Early Warning Score 2 | Routinely allocates recorded scores of physiological parameters (e.g. respiration rate, oxygen saturation, systolic blood pressure, pulse rate, level of consciousness/new confusion, and temperature), with a cut-off of ≥ 5 points for clinical deterioration in acutely ill patients (52, 53). |
| Clinical outcomes (assessed pre-post interventions and at discharge) | | |
| 1. Trajectory of delirium symptoms: | DSM-5 criteria | DSM-5 diagnostic algorithm and test battery, comprising validated scales and tests for evaluating each DSM-5 criteria (37). |
| \| AROUSAL \| \| --- \| \| ATTENTION \| \| ORIENTATION  and  SHORT TERM MEMORY \| | \| OSLA: Observational Scale of Level of Arousal \| \| --- \| \| mRASS: Modified Richmond Agitation Sedation Scale  ‘ \| \| Digit span test \| \| Backwards tests:   - Months of the year - Days of the week - Counting 20-1 \| \| Vigilance test (SAVEHAART) \| \| Recall tasks  Orientation questions \| | \| OSLA is a reliable and validated instrument for assessing arousal by observing eye opening, eye contact, posture and movement, and the scores range from 0 to 15 (42). The score ≥ 3 is the cut-off for abnormal arousal, for which OSLA scale has sensitivity of 0.85 (95% CI [72-93]), and specificity of 0.82 (95% CI [71-91] (42). \| \| --- \| \| mRASS measures changes in sedation and agitation by observing the duration of eye contact following verbal and physical stimulation (55, 56). mRASS has strong validity and reliability in geriatric and critically ill populations (57). The scores range from −5 (comatose state) to +5 (combativeness), with negative scores indicating more hypo-aroused states, and positive scores indicating hyper-arousal (56). With a cut-off ≠ 0 for abnormal sedation/agitation, the sensitivity of the mRASS scale is 0.90 (95% CI [56-100]), and the specificity 0.85 (95% CI [62-97]). \| \| The digit span test has scores ranging between 0-7. \| \| - Months of the Year Backwards (MOYB) has scores ranging from 0-12. - Days of the Week Backwards (DOWB) has scores ranging between 0-7. - Backwards counting from 20-1 has scores ranging from 0-20 (41, 44). \| \| A ten-letter vigilance “A” task (SAVEHAAART) is taken from the Confusion Assessment Method for the Intensive Care Unit (CAM-ICU) scale and has scores ranging from 0-4 (41, 44). \| \| - The orientation test consisted of 10 predefined questions for the patients (ranging from 0-10) - Short term memory and delayed recall test involved repeating 3 words after a series of other tasks and had scores ranging from of 0-3(58).   Orientation, short-term memory, and recall tests originate from the validated MDAS scale assessing the severity of delirium (58). Cut-offs of the tests indicating inattention, disorientation, and impaired short-term memory may be found in Appendix 2 and in our published protocol (37) \| |
| 1. Duration of delirium | DSM-5 assessments  Medical journals | N.R. |
| 1. Length of hospital stay | Electronic medical journals | N.R. |
| 1. Use of PRN medication | Electronic medical journals | N.R. |
| Feasibility outcomes | | |
| Treatment fidelity | Author-developed checklists for PLM and PRM. | The six checklist items (no = 0, yes = 1 point) were calculated, and the threshold for satisfied treatment fidelity for each participant was ≥80 % averaged across the three intervention days, including satisfied compulsory items 4-6 for each session. The intervention was considered not to have met fidelity if the compulsory items were not satisfied even if the total score was ≥80 %. |
