# Supplementary material for: A randomized pilot and feasibility trial of live and recorded music interventions for management of delirium symptoms in acute geriatric patients
Source: BMC Geriatr. 2025 May 2;25:306. doi: 10.1186/s12877-025-05954-1 (PMC12048927; doi:10.1186/s12877-025-05954-1)
Supplement: Supplementary file 2 — Additional file 2. Diagnostic algorithm for DSM-5 delirium evaluation. [file 12877_2025_5954_MOESM2_ESM.docx]

**Additional file 2.** Diagnostic algorithm for DSM-5 delirium evaluation

| **DSM-5 Criteria** | **Tests to be performed and information to be collected** | **Is DSM-criteria fulfilled?** | |
| --- | --- | --- | --- |
|  |  | YES | NO |
| A. Disturbance in **attention** (i.e. reduced ability to direct, focus, sustain, and shift attention) and **awareness** (reduced orientation to the environment | \| Evaluation \| Attention-tests \| Cut off (definition of inattention) \| \| --- \| --- \| --- \| \| Daily \| SAVEAHAART/ KATAMARAAN \| 2 or more errors \| \| Days of the week in reversed order \| Any error \| \| Months of the year in reverse order \| Unable to reach July \| \| Count backwards from 20 to 1 \| Any error \| \| Digit span forward \| Less than 5 forward \|   Observation:  Easily distracted? Collaborative? Has a tendency to “loose thread” in the conversation? |  |  |
|  | Arousal: OSLA >3 and/or mRASS other than 0? |  |  |
| B. The disturbance develops over a **short period of tim**e (usually hours to a few days), represents **a change** from baseline attention and awareness, and **tends to fluctuate** in severity during the course of a day. | Informant history from patient’s carers and nursing staff. Questions to carer/nursing staff or derived from clinical notes:   - Has there been a sudden change in the patient’s mental state? - Does the patient seem to be better at any period in the day compared to other times? - Has the level of consciousness been altered (drowsy/not responsive, or agitated)? - Sleep-wake cycle disturbances? |  |  |
| C. An additional **disturbance in cognition** (e.g. memory deficit, disorientation, language, visuospatial ability, or perception). | Questions to the patients:  Orientation-tests*:* Orientation to time, place and person; Why are you in hospital? Will a stone float in water? Are there fish in the sea? (any error=disorganized thinking)  Recall (3 words)  Questions to carers/nursing staff/clinical notes:  Has there been any…Perceptual disturbances? Sleep-wake cycle disturbances? Memory disturbances? Psychotic episodes? Psychomotor disturbances? |  |  |
| D. The disturbances in criteria A and C are not explained by another preexisting, established | Information from history/chart/clinical assessment. |  |  |
| E. There is evidence from the history, physical examination, or laboratory findings that the disturbance is a direct physiological consequence of another medical condition, substance intoxication or withdrawal (i.e., due to a drug of abuse or to a medication), or exposure to a toxin, or is due to multiple aetiologies. |  |  |  |
| Delirium, based on the tests and information above? | All DSM-5 criteria are fulfilled |  |  |
| Subsyndromal delirium, based on the tests and information above? | Defined as evidence of change, in addition to any of the following: (a) altered arousal, (b) attention deficits, (c) other cognitive change, (d) delusions or hallucinations.  Criteria D and E must be fulfilled. |  |  |
