# Supplementary material for: A randomized pilot and feasibility trial of live and recorded music interventions for management of delirium symptoms in acute geriatric patients
Source: BMC Geriatr. 2025 May 2;25:306. doi: 10.1186/s12877-025-05954-1 (PMC12048927; doi:10.1186/s12877-025-05954-1)
Supplement: Supplementary file 3 — Additional file 3. Checklists for treatment fidelity evaluation (PLM, PRM). [file 12877_2025_5954_MOESM3_ESM.docx]

**Additional file 3**. Checklists for treatment fidelity evaluation (PLM, PRM)

| **PREFERRED LIVE MUSIC (PLM)** | | | | | |
| --- | --- | --- | --- | --- | --- |
| **Study ID:** | | **Intervention day:** | **Criteria satisfied** | | **Score** |
| 1. | MT delivered the intended minimum dosage (participant was in attendance for 10 min or longer). | | YES | NO |  |
| 2. | MT used the assessed preference songs in the session. * | | YES | NO |  |
| 3. | MT presented the preference songs in the same order as intended/planned (relevant for sessions 2 and 3 only) | | YES | NO |  |
| 4. | MT delivered the intervention by voice only, or voice and accompaniment (e.g. guitar, tone-chimes, percussions). | | YES | NO |  |
| 5. | MT cued the participant to sing along or move to music, used the elements of improvisation and attunement to the patient. ** | | YES | NO |  |
| 6. | MT had physical (holding hands, stroking, etc.), verbal, or non-verb verbal interaction with the participant (eye-contact, face expressions, smiling). | | YES | NO |  |
| Total: | | | | |  |

Intervention manual:

*In PLM intervention music therapist should chose the music for the sessions from patients’ assessed preferences and make sure not to change their order of deliverance. MT should enter the room, say hi to the patient, introduce the intervention, and if appropriate, offer the participants small percussion instruments that they can play on during the session. Thereafter MT should deliver the intervention live, by singing/playing the songs either A Capella, or accompanied by a guitar, tone-chimes, or percussion instruments. The intervention may involve interaction with the patients (e.g. physical, musical, verbal), the songs are not expected to be delivered in a way that is identical to their original versions, and elements of improvisation are both allowed and expected, as well as other forms of attunement to the patients. After max. 30 minutes, the MT should collect the instruments, say good bye to the patient, and leave the room.*

Instructions for scoring:

YES: I agree with the statement (score 1)

NO: I disagree with the statement (score 0)

NR: Not relevant

* Item 2 is only relevant for sessions 2 and 3 and not relevant for sessions 1. Should be scored as NR.

** Item 5 is related to the presence of improvisation elements, such as repetition, variation, extension, mirroring, matching, imitation, etc. as well as other musical or non-musical elements and forms of attunement to the patient (cuing, humming, whistling, scatting, snapping fingers, laughing together etc. The rater should score YES if there is at least one of the aforementioned elements present, and as long as there is a minimum variation in the performance of the songs compared to the original.

| **PREFERRED RECORDED MUSIC (PRM)** | | | | | | |
| --- | --- | --- | --- | --- | --- | --- |
| **Study ID:** | | **Intervention day:** |  | **Criteria satisfied:** | | **Score** |
| 1. | MT delivered the intended minimum dosage (participant was in attendance for the minimum of 10 min). | | | YES | NO |  |
| 2. | MT used the assessed preference songs in the session. * | | | YES | NO |  |
| 3. | MT presented the preference songs in the same order as intended/planned (relevant for sessions 2 and 3 only). | | | YES | NO |  |
| 4. | MT delivered the intervention by the speaker and Bluetooth musical device. | | | YES | NO |  |
| 5. | MT did not cue the participant to sing along or move to music. | | | YES | NO |  |
| 6. | MT did not have physical (holding hands, stroking, etc.), verbal, or non-verb verbal interaction with the participant (eye-contact, face expressions, smiling etc.). | | | YES | NO |  |
| Total: | | | | | |  |

Intervention manual:

*In the PRM intervention, music therapist (MT) should chose the songs for the sessions from the patients’ assessed preferences (and not vary their order each of the three intervention days). MT should enter the participant’s room, and after saying hi and shortly introducing the music that will be played, start the music from a musical device and a Bluetooth speaker. MT should refrain from engaging with the patient while the music is played, and any engagement should be registered as the disruption from the protocol. The MT should not engage with the patient neither verbally, non-verbally, physically while the music is played. Any engagement should be registered as the disruption of the protocol. After 30 minutes the MT should stop the music, say good bye to the patient and leave the room.*

Instructions for scoring:

YES: I agree with the statement (score 1)

NO: I disagree with the statement (score 0)

NR: Not relevant

*Item 2 was only relevant for sessions 2 and 3. Should be scored as NR for session 1.
