# Supplementary material for: A randomized pilot and feasibility trial of live and recorded music interventions for management of delirium symptoms in acute geriatric patients
Source: BMC Geriatr. 2025 May 2;25:306. doi: 10.1186/s12877-025-05954-1 (PMC12048927; doi:10.1186/s12877-025-05954-1)
Supplement: Supplementary file 4 — Additional file 4. Group PLM versus Group PRM difference on Day 3. [file 12877_2025_5954_MOESM4_ESM.docx]

**Additional file 4.** Group PLM versus Group PRM difference on Day 3

| **Measure** | **Before/after** | **PLM** | **PRM** | **Mean difference (95 % CI)** | **p-value** |
| --- | --- | --- | --- | --- | --- |
| OSLA | Before | 3.0 (1.4 to 4.5) | 4.1 (1.4 to 6.9) | 1.2 (-2.0 to 4.3) | 0.463 |
|  | After | 3.9 (2.3 to 5.5) | 2.8 (0.0 to 5.6) | -1.1 (-4.3 to 2.1) | 0.514 |
| mRASS | Before | -0.5 (-0.9 to 0) | -0.4 (-1.2 to 0.4) | 0.1 (-0.9 to 1) | 0.874 |
|  | After | -0.9 (-1.4 to -0.4) | -0.1 (-0.9 to 0.7) | 0.7 (-0.2 to 1.7) | 0.125 |
| Count 20 to 1 | Before | 14.1 (9.8 to 18.4) | 13.6 (6.8 to 20.3) | -0.6 (-8.5 to 7.4) | 0.890 |
|  | After | 11.8 (7.3 to 16.2) | 9.6 (2 to 17.2) | -2.2 (-11 to 6.6) | 0.625 |
| Days of the week | Before | 5.4 (3.9 to 7.0) | 5.4 (3.0 to 7.8) | -0.1 (-2.9 to 2.8) | 0.972 |
|  | After | 5.3 (3.6 to 6.9) | 3.9 (1.5 to 6.3) | -1.4 (-4.3 to 1.6) | 0.358 |
| Months of the year | Before | 4.3 (2.3 to 6.3) | 1.8 (-1.3 to 4.9) | -2.4 (-6.1 to 1.3) | 0.195 |
|  | After | 4.7 (2.6 to 6.8) | 3.3 (0.0 to 6.8) | -1.5 (-5.5 to 2.6) | 0.485 |
| Digit span | Before | 3.9 (2.9 to 4.9) | 5.2 (3.6 to 6.7) | 1.2 (-0.6 to 3.1) | 0.197 |
|  | After | 3.5 (2.4 to 4.5) | 3.9 (2.3 to 5.5) | 0.4 (-1.5 to 2.3) | 0.657 |
| SAVEAHEART | Before | 1.9 (0.0 to 3.7) | 1.6 (0.0 to 4.4) | -0.3 (-3.6 to 3.1) | 0.881 |
|  | After | 2.6 (0.7 to 4.5) | 1.3 (0.0 to 4.2) | -1.3 (-4.7 to 2.1) | 0.458 |
| Orientation | Before | 5.0 (3.7 to 6.3) | 4.3 (2.3 to 6.4) | -0.7 (-3.1 to 1.7) | 0.576 |
|  | After | 4.0 (2.7 to 5.4) | 4.4 (2.0 to 6.7) | 0.3 (-2.4 to 3.0) | 0.813 |

*PLM* Preferred Live Music, *PRM* Preferred Recorded Music

^a^ The estimated confidence intervals from the linear mixed models had lower limits below zero, however the scores do not go below zero and the lower limit has therefore been adjusted to zero.
