# Supplementary material for: A randomized pilot and feasibility trial of live and recorded music interventions for management of delirium symptoms in acute geriatric patients
Source: BMC Geriatr. 2025 May 2;25:306. doi: 10.1186/s12877-025-05954-1 (PMC12048927; doi:10.1186/s12877-025-05954-1)
Supplement: Supplementary file 5 — Additional file 5. Proportion of participant who could recall any words in delayed recall test. [file 12877_2025_5954_MOESM5_ESM.docx]

**Additional file 5**. Proportion of participant who could recall any words in delayed recall test

| Day | Before/after | Proportion (95 % CI) |
| --- | --- | --- |
| Baseline |  | 19.2 (8.2 to 38.9) |
| Day 1 | before | 9.1 (2.3 to 30.2) |
| Day 1 | after | 15.0 (4.9 to 37.9) |
| Day 2 | before | 15.8 (5.1 to 39.4) |
| Day 2 | after | 11.8 (2.9 to 37.2) |
| Day 3 | before | 20.0 (6.5 to 47.3) |
| Day 3 | after | 7.7 (1.1 to 39.5) |
