# Supplementary material for: A randomized pilot and feasibility trial of live and recorded music interventions for management of delirium symptoms in acute geriatric patients
Source: BMC Geriatr. 2025 May 2;25:306. doi: 10.1186/s12877-025-05954-1 (PMC12048927; doi:10.1186/s12877-025-05954-1)
Supplement: Supplementary file 6 — Additional file 6. Between the groups difference in Length of hospital stay and PRN medication. [file 12877_2025_5954_MOESM6_ESM.docx]

**Additional file 6.** Between the groups difference in Length of hospital stay and PRN medication

| **Variable** | **PLM (n=14)** | **PRM (n=12)** | **Mann-Whitney test *(U*)** | **Significance** *(p)* |
| --- | --- | --- | --- | --- |
| Length of hospital stay,  Mean (SD) ^a^ | 11 (8.95) | 13 (9.94) | 82.500 | 0.940 |
| Number of patients receiving PRN medication during hospital stay,  n (%) ^b^ | | | **Fisher Exact test *(p)*** | |
| Benzodiazepines^b^ | 7 (43) | 4 (33) | 0.431 | |
| Opioids^b^ | 9 (64) | 6 (50) | 0.422 | |
| Antipsychotics^b^ | 5 (36) | 2 (17) | 0.286 | |

*PLM* Preferred Live Music, *PRM* Preferred Recorded Music *PRN* Pro-re-nata, psychopharmacological “rescue” medication.

^a^ Mean ranks

^b^ Number and percentage of patients who got the PRN medication during hospital stay
